# Editorial: the 20th annual *Nucleic Acids Research* Web Server Issue 2022

**DOI:** 10.1093/nar/gkac525

**Published:** 2022-07-05

**Authors:** Dominik Seelow

**Affiliations:** Exploratory Diagnostic Sciences, Berliner Institut für Gesundheitsforschung, Berlin 10117, Germany; Institut für Medizinische Genetik und Humangenetik, Charité – Universitätsmedizin Berlin, corporate member of Freie Universität Berlin and Humboldt-Universität zu Berlin, Berlin 13353, Germany

This is the 20th edition of the *Nucleic Acids Research* Web Server Issue. Under normal circumstances, this would be an opportunity to celebrate our success as the leading source for peer-reviewed web servers. However, circumstances are far from normal and in a time of pandemic and conflict, my thanks go to all those who have helped others, including many of our authors and referees. Indeed, some of our authors even risked missing the slot for publication in this year's Issue by deciding to rather care for others. Thank you!

And so, to this edition of the Web Server Issue. We received 301 proposals describing new or updated web servers and stand-alone tools this year. One hundred thirty-three were invited to submit a manuscript, 102 (77%) of the submissions were accepted, leading to an acceptance rate of one third of the initial proposals. Nine proposals were handled by different editors because I had a conflict of interest. A big thank you to Janusz Bujnicki, Mair Churchill, Alan Kimmel, Dan Rigden, Julian Sale and Barry Stoddard for your help.

Another—even bigger—thank you goes to our internal in-depth reviewers:

- **Georges Schmartz**, PhD student, Universität des Saarlandes, Saarbrücken, Germany
- **Viktoria Wagner**, PhD student, Universität des Saarlandes, Saarbrücken, Germany
- **Adam Simpkin**, PhD, University of Liverpool, UK
- **Lennard Ostendorf**, MD & PhD, Charité – Universitätsmedizin Berlin, Germany

The in-depth review is an important step to test web servers for function and user-friendliness, including thorough documentation, example data, and a tutorial. We invite authors only to submit a manuscript if this review leads to a positive report. Direct manuscript submissions are not considered without this validation.

As in recent years, the vast majority of proposals was submitted just before the deadline on 20 December. I would like to use this opportunity to encourage you to submit your proposals as early as possible—sifting through hundreds of proposals and performing in-depth testing of the websites is time-consuming and a more balanced inflow would help us a lot. An earlier invitation to submit a manuscript would also give you more time to prepare it.

One common reason for rejection was the lack of user-friendliness. This became particularly apparent for stand-alone software, a special category within the Web Server Issue. It is—or rather should be—easy to use a web server and we expect stand-alone tools to be usable without the requirement to install many third-party applications or to up or downgrade to a specific Java or Python version.

Another frequent problem is the application of new methods. The Web Server Issue is aimed at useful, usable, and user-friendly web servers, it is not intended to publish new method. Due to our fixed publication date, we have very short turn-around times for the reviews and this time would often not suffice to validate a method.

One key requirement for Web Server Issue publications is free access to the web server without limitations (including commercial use), without the requirement to register or to provide an email address. This edition was the first to allow a login requirement if—**and only if**—users can access sensitive data (e.g. human WES or medical data). We still do not have a general requirement to use SSL encryption (https) for non-sensitive data but we would like to encourage you to use https when developing web servers.

Another change is the cookie policy: While we still allow cookies, websites must now provide a cookie consent form or at least a cookie notification. Tracking cookies must not be used.

The 2022 Web Server Issue contains the following 102 applications:

**Table:** 

Short title	URL	short description
3DGenBench	https://inc-cost.eu/benchmarking/	benchmarking computational models for 3D genomics
3DLigandSite	https://www.wass-michaelislab.org/3dligandsite	structure-based prediction of protein-ligand binding sites
5NosoAE	https://nosoae.imst.nsysu.edu.tw	nosocomial bacterial antibiogram investigation and epidemiology survey
AlignMe	https://www.bioinfo.mpg.de/AlignMe/	alignment of membrane protein sequences
AllerCatPro 2.0	https://allercatpro.bii.a-star.edu.sg	predict protein allergenicity potential
AlphaKnot	https://alphaknot.cent.uw.edu.pl/	analyse entanglement in structures predicted by AlphaFold methods
ANANASTRA	https://ananastra.autosome.org	annotation and enrichment analysis of allele-specific transcription factor binding
Annotation Query	http://annoq.org/	annotation of genetic vairants
Aquaculture Molecular Breeding Platform	http://mgb.qnlm.ac	genetic data analysis in aquatic species
AutoESD	https://autoesd.biodesign.ac.cn/	genetic manipulation of microorganisms
AutozygosityMapper	https://www.genecascade.org/AutozygosityMapper/	identification of disease-mutations in consanguineous families
BeStSel	https://bestsel.elte.hu	secondary structure and fold prediction for protein CD spectroscopy
BioBB-Wfs	https://mmb.irbbarcelona.org/biobb-wfs	BioExcel Building Blocks Workflows for biomolecular simulations
BioSimulators	https://biosimulators.org	registry of simulation tools
BioTransformer 3.0	https://biotransformer.ca	prediction of metabolic transformation products
BioUML	https://www.biouml.org	platform for systems biology and data analysis
BusyBee Web	https://www.ccb.uni-saarland.de/busybee	genomic binning for metagenomic data analysis
CADDIE	https://exbio.wzw.tum.de/caddie/	Cancer Driver Drug Interaction Explorer
CalFitter 2.0	https://loschmidt.chemi.muni.cz/calfitter2	protein thermostability
CATANA	http://catana.ait.ac.at/	modelling environment for proteins and nucleic acid nanostructures
CB-Dock2	https://cadd.labshare.cn/cb-dock2/	predicting protein-ligand blind docking by integrating cavity detection
CFM-ID 4.0	https://cfmid.wishartlab.com	mass spectrometry-based metabolite identification
ChIP-Atlas 2021	https://chip-atlas.org	data-mining suite for exploring epigenomic landscapes
CircadiOmics	http://circadiomics.igb.uci.edu/	portal for high-throughput circadian time series data
CRISPRedict	http://www.crispredict.org/	CRISPR-Cas9 efficiency predictions
CRISPRnano	https://www.crisprnano.de/	identification of genome-edited cells
CSM-potential	http://biosig.unimelb.edu.au/csm_potential	mapping protein interactions and biological ligands in 3D space
Dali	http://ekhidna2.biocenter.helsinki.fi/dali	unification of protein families by structure comparison
DAVID	https://david.ncifcrf.gov	functional enrichment analysis and annotation of gene lists
DDGun	http://folding.biofold.org/ddgun	prediction of protein stability changes upon amino acid variants
DeepLoc 2.0	https://services.healthtech.dtu.dk/service.php?DeepLoc-2.0	prediction of protein subcellular localization
DEMO2	https://zhanggroup.org/DEMO/	multi-domain protein structure assembly
DEPCOD	http://genetics.mgh.harvard.edu/DEPCOD	protein domain co-evolution detection and visualization
DLEB	http://dleb.konkuk.ac.kr/	creation of deep learning models for biological research
DNAzymeBuilder	https://iimcb.genesilico.pl/DNAzymeBuilder	in situ generation of RNA/DNA-cleaving deoxyribozymes
DREEM	http://rnadreem.org	analysis of RNA folding from high throughput chemical probing data
DrugVirus.info 2.0	https://drugvirus.info	data portal for broad-spectrum antivirals
EBI Search & Sequence Analysis	https://www.ebi.ac.uk/ebisearch	EMBL-EBI search and sequence analysis Ttools
enrichMiR	https://ethz-ins.org/enrichMiR/	prediction of functionally relevant microRNAs
EPIXplorer	https://www.csuligroup.com/EPIXplorer	prediction, analysis & visualization of enhancer-promoter interactions
ERMer	https://ermer.biodesign.ac.cn	analyse and visualize the *Escherichia coli* regulatory landscape
ExPheWas	https://exphewas.ca	*cis*-Mendelian randomization and gene-based association
ExpressVis	https://omicsmining.ncpsb.org.cn/ExpressVis	visual exploration of multi-omics data
FABIAN-variant	https://www.genecascade.org/FABIAN/	predicting the effects of DNA variants on transcription factor binding
FUNAGE-Pro	http://funagepro.molgenrug.nl	gene set enrichment analysis for prokaryotes
FuzDrop on AlphaFold	https://fuzdrop.bio.unipd.it	prediction of liquid-liquid phase separation and aggregation of proteins
Galaxy	https://galaxyproject.org	Galaxy platform 2022 update
GeCoViz	https://gecoviz.cgmlab.org	exploration of genomic context conservation of prokaryotic genes
GenePlexus	https://www.geneplexus.net/	gene discovery using network-based machine learning
GEOexplorer	https://geoexplorer.rosalind.kcl.ac.uk/	gene expression analysis and visualization
GrAfSS	http://mfrlab.org/grafss/	substructure similarity searching for proteins and RNA
GraPES	https://grapes.msl.ubc.ca/	prediction of protein localization into cellular condensates
GRaSP-web	https://grasp.ufv.br	prediction of protein binding sites
GRNbenchmark	https://grnbenchmark.org/GRNbenchmark	benchmarking directed gene regulatory network inference methods
HemI 2.0	https://hemi.biocuckoo.org/	heatmap illustration
iBIS2Analyzer	http://ibis2analyzer.lcqb.upmc.fr/	phylogeny-driven co-evolution analysis of protein families
IBS 2.0	https://ibs.renlab.org	illustrator for biological sequences
ICARUS	https://launch.icarus-scrnaseq.cloud.edu.au	single cell RNA-seq analysis
iFeatureOmega	http://ifeatureomega.erc.monash.edu	feature generation and analysis for sequences, structures & ligands
KmerKeys	https://kmerkeys.dgi-stanford.org/	fast search for *k*-mers in genomes
LOMETS3	https://zhanglab.ccmb.med.umich.edu/LOMETS/	template-based protein structure prediction and function annotation
LoopGrafter	https://loschmidt.chemi.muni.cz/loopgrafter/	transplantation of loops between structurally-related proteins
MAPIYA	http://mapiya.lcbio.pl/	identification and visualization of molecular interactions in proteins and biological complexes
MDsrv	https://proteinformatics.informatik.uni-leipzig.de/mdsrv	visual sharing and analysis of molecular dynamics simulations
mitoXplorer 2.0	http://mitoxplorer2.ibdm.univ-mrs.fr	mitochondrial expression dynamics
Multi-CSAR	http://genome.cs.nthu.edu.tw/Multi-CSAR/	scaffolding contigs using multiple reference genomes
NetSurfP-3.0	https://dtu.biolib.com/nsp3	prediction of protein structural features by protein language models
Ocean Gene Atlas v2.0	https://tara-oceans.mio.osupytheas.fr/ocean-gene-atlas/	biogeography and phylogeny of plankton genes
OmicsNet 2.0	https://www.omicsnet.ca/	multi-omics integration and network visual analytics
OrthoQuantum	http://orthoq.bioinf.fbb.msu.ru	visualizing the evolutionary repertoire of eukaryotic proteins
PADLOC	https://padloc.otago.ac.nz/	identification of antiviral defence systems in microbial genomes
PaintOmics4	https://paintomics.org/	pathway-based analysis of multi-omics datasets
patcHwork	https://patchwork.biologie.uni-freiburg.de/	pH sensitivity analysis of protein sequences and structures
PCGA	https://pmglab.top/pcga	phenotype-cell-gene association analysis
PhyloCloud	https://phylocloud.cgmlab.org	tools for phylogenomics
PiER	http://www.genetictargets.com/PiER	target prioritization for genetic association studies
PrankWeb 3	https://prankweb.cz/	protein-ligand binding site prediction
PRECOGx	https://precogx.bioinfolab.sns.it/	exploring GPCR signalling mechanisms
ProteinsPlus	https://proteins.plus	protein modelling tools focusing on protein-ligand interactions
pubmedKB	https://www.pubmedkb.cc/	exploring biomedical entity relations in the biomedical literature
Quest for Orthologs 2022	https://orthology.benchmarkservice.org	orthology benchmark service
RaacFold	http://bioinfor.imu.edu.cn/raacfold	visualization and analysis of protein structure by using reduced amino acid alphabets
REVERSE	https://www.reverseserver.org/	analysing next-generation sequencing data from *in vitro* selection/evolution experiments
RING 3.0	https://ring.biocomputingup.it/	probabilistic residue interaction network generation
rna-tools.online	https://rna-tools.online	RNA 3D structure modelling tools
RNAspider	https://rnaspider.cs.put.poznan.pl	analyse entanglements in RNA 3D structures
RSAT 2022	http://www.rsat.eu/	Regulatory Sequence Analysis Tools
SAMS	https://www.genecascade.org/SAMS/	sign-based deep phenotyping
SeMa-Trap	https://sema-trap.ziemertlab.com	target gene prioritization for biosynthetic gene cluster overexpression
Shiny GATOM	https://ctlab.itmo.ru/shiny/gatom	omics-based identification of regulated metabolic modules in atom transition networks
SigCom LINCS	https://maayanlab.cloud/sigcom-lincs	drug and target discovery using gene expression signatures
sRNAbench / sRNAtoolbox 2022	https://arn.ugr.es/srnatoolbox/	miRNA and sncRNA profiling
SubcellulaRVis	http://phenome.manchester.ac.uk/subcellular/	subcellular compartment enrichment
SuperPred 3.0	https://prediction.charite.de/index.php	drug classification and target prediction
SWORD2	https://www.dsimb.inserm.fr/SWORD2/	hierarchical analysis of protein 3D structures
SynergyFinder 3.0	https://synergyfinder.fimm.fi	multi-drug and multi-sample synergy analyses
TADeus2	https://tadeus2.mimuw.edu.pl	pathogenicity prediction for structural variants
TeachOpenCADD 2022	https://projects.volkamerlab.org/teachopencadd	pipelines for drug discovery
TIRSF	http://tirsf.renlab.org/	predicting tumour immunotherapy response using gene signatures
VRprofile2	https://tool2-mml.sjtu.edu.cn/VRprofile	antibiotic resistance-associated mobilome in bacterial pathogens
WashU Epigenome Browser 2022	https://epigenomegateway.wustl.edu/browser/	visualization, integration & analysis of epigenomic datasets
WebCSEA	https://bioinfo.uth.edu/webcsea/	cell type specific gene enrichment analysis

I hope that you will find many of them useful.

With this, it is time to thank the wonderful team at Oxford University Press for their help. Most of all (as always) Martine Bernardes Silva and Tina Belle who has recently joined the team. Without your support, there would not have been any Web Server Issue at all.

Our reviewers also deserve my gratitude. The Web Server Issue requires very short turn-around times and I know how challenging it is to provide a thorough review. Especially when it is not only a manuscript to be evaluated, but also a web server. Thank you so much for taking the time.

Should you wish to submit a manuscript to the 2023 Web Server Issue, please check https://academic.oup.com/nar/pages/submission_webserver for any changes and try to submit your proposal as early as possible. The whole team at *NAR* – and the scientific community—is looking forward to new and exciting web servers and updates of existing tools.

Dominik Seelow

*NAR* Web Server Issue Executive Editor

